# Alteration in branching morphogenesis via YAP/TAZ in fibroblasts of fetal lungs in an LPS-induced inflammation model

**DOI:** 10.1186/s10020-023-00613-w

**Published:** 2023-01-30

**Authors:** Hung-Shuo Ko, Vincent Laiman, Po-Nien Tsao, Chung-Ming Chen, Hsiao-Chi Chuang

**Affiliations:** 1 School of Medicine, College of Medicine, Taipei Medical University, Taipei, Taiwan; 2 International Ph.D. Program in Medicine, College of Medicine, Taipei Medical University, Taipei, Taiwan; 3 Department of Anatomical Pathology, Faculty of Medicine, Public Health, and Nursing, Dr. Sardjito Hospital, Universitas Gadjah Mada, Yogyakarta, Indonesia; 4 Department of Pediatrics, National Taiwan University Hospital, Taipei, Taiwan; 5 Department of Pediatrics, Taipei Medical University Hospital, Taipei, Taiwan; 6 Department of Pediatrics, School of Medicine, College of Medicine, Taipei Medical University, Taipei, Taiwan; 7 School of Respiratory Therapy, College of Medicine, Taipei Medical University, 250 Wuxing Street, Taipei, 11031 Taiwan; 8 Division of Pulmonary Medicine, Department of Internal Medicine, Shuang Ho Hospital, Taipei Medical University, New Taipei City, Taiwan; 9 Cell Physiology and Molecular Image Research Center, Wan Fang Hospital, Taipei Medical University, Taipei, Taiwan; 10 Graduate Institute of Medical Sciences, College of Medicine, Taipei Medical University, Taipei, Taiwan; 11 National Heart & Lung Institute, Imperial College London, London, UK

**Keywords:** Fibroblast, Hippo signaling pathway, Inflammation, Morphogenesis, Pseudoglandular stage

## Abstract

**Background:**

Chorioamnionitis is a common cause of preterm birth and leads to serious complications in newborns. The objective of this study was to investigate the role of the Hippo signaling pathway in lung branching morphogenesis under a lipopolysaccharide (LPS)-induced inflammation model.

**Materials and methods:**

IMR-90 cells and ex vivo fetal lungs were treated with 0, 10, 30, or 50 μg/ml LPS for 24 and 72 h. Supernatant levels of lactate dehydrogenase (LDH), interleukin (IL)-6, IL-8, Chemokine (C-X-C motif) ligand 1(CXCL1), branching and the surface area ratio, Yes-associated protein (YAP), transcription coactivator with PDZ-binding motif (TAZ), fibroblast growth factor 10 (FGF10), fibroblast growth factor receptor II (FGFR2), SRY-box transcription factor 2 (SOX2), SOX9, and sirtuin 1 (SIRT1) levels were examined. Differentially expressed genes in fetal lungs after LPS treatment were identified by RNA-sequencing.

**Results:**

LPS at 50 μg/ml increased IL-6 and IL-8 in IMR-90 cells and increased IL-6, CXCL1 and LDH in fetal lungs. The branching ratio significantly increased by LPS at 30 μg/ml compared to the control but the increased level had decreased by 50 μg/ml LPS exposure. Exposure to 50 μg/ml LPS increased phosphorylated (p)-YAP, p-YAP/YAP, and p-TAZ/TAZ in IMR-90 cells, whereas 50 μg/ml LPS decreased FGF10 and SOX2. Consistently, p-YAP/YAP and p-TAZ/TAZ were increased in fibronectin^+^ cells of fetal lungs. Moreover, results of RNA-sequencing in fetal lungs showed that SMAD, FGF, IκB phosphorylation, tissue remodeling and homeostasis was involved in branching morphogenesis following exposure to 50 μg/ml LPS. The p-SIRT1/SIRT1 ratio increased in IMR-90 cells by LPS treatment.

**Conclusions:**

This study showed that regulation of the Hippo pathway in fibroblasts of fetal lungs was involved in branching morphogenesis under an inflammatory disease such as chorioamnionitis.

**Supplementary Information:**

The online version contains supplementary material available at 10.1186/s10020-023-00613-w.

## Introduction

Distributing the air to the gas-exchange zone of the lungs is achieved by the conducting airways in mature lungs. Formation of this tree-like system is mostly performed in the pseudoglandular stage which occurs from embryonic day 10.5 (E10.5) to E16.5 in mice and 6 to 16 weeks post-menstrual age (PMA) in humans (Smith et al. 2010). Two primary buds undergo a branching process to establish the airway structure, and nearly 20 generations of future airways are completed (Kitaoka et al. 1996). Impairment of branching morphogenesis was associated with lung hypoplasia which accounts for approximately 7–26% of neonatal autopsies (Husain and Hessel 1993), resulting in high rates of morbidity and mortality in newborns, such as congenital diaphragmatic hernias (Coughlin et al. 2016). Previous studies showed that lung branching was related to antenatal exposure to inflammatory mediators such as interleukin (IL)-6 and IL-8 (Dame and Juul 2000; Nogueira-Silva et al. 2006). Chorioamnionitis is defined as inflammation of the membrane and chorion of the placenta, which is known to be a frequent cause of preterm births (Tita and Andrews 2010). A dramatic increase in IL-6 in the amniotic fluid was observed in chorioamnionitis patients (Tsuda et al. 1998). However, the underlying mechanism regarding branching morphogenesis under chorioamnionitis is still unknown.

Chorioamnionitis, the most common type of antenatal inflammation, predominantly presents with intra-amniotic inflammation but with no evidence of microbial invasion (Kunzmann et al. 2013). Several risk factors were identified for chorioamnionitis, including bacterial vaginosis, group B streptococci, alcohol and tobacco use (Tita and Andrews 2010). Inflammatory mediators are first induced by a maternal immune response, followed by fetal inflammatory response syndrome, which causes the fetus to develop serious complications (Gomez et al. 1998; Higgins et al. 2016). A meta-analysis reported that chorioamnionitis was associated with a decreased risk of respiratory distress syndrome (RDS) and increased risk of bronchopulmonary dysplasia (BPD) (Sarno et al. 2021). Therefore, chorioamnionitis causes lung maturation and injury, and increases the risk of chronic lung diseases (CLDs) in preterm infants.

Fibroblasts are vital to the development of all stages of the lungs. Interactions between epithelial cells and fibroblasts rely on the proximity to the epithelium (Caniggia et al. 1991). During the pseudoglandular stage, fibroblasts stimulate cell proliferation in the lung epithelium, while promoting cell differentiation in the saccular stage (Caniggia et al. 1992). The formation and remodeling of the extracellular matrix (ECM) via matrix-fibroblasts, lipofibroblasts, and myofibroblasts in the process of lung development create tensile strength (Ushakumary et al. 2021). Lipofibroblasts are capable of taking up triglycerides, which support the synthesis of surfactants in type 2 alveolar epithelial cells (Torday and Rehan 2016), and they were also demonstrated to protect the lungs from hyperoxic injury (Rehan et al. 2006). Myofibroblasts play a key role in alveologenesis through activating platelet-derived growth factor (PDGF)/PDGF receptor (PDGFR)-α signaling, which was validated by inactivating the pathway which suspended alveolarization in mice (Boström et al. 2002; Boström et al. 1996). Recently, alveolar niche cells were categorized as fibroblasts and were observed to support alveolar epithelial regeneration after injury (Zepp et al. 2017).

The Hippo signaling pathway is considered to be crucial for lung development (Fu et al. 2017). The Yes-associated protein (YAP) and transcriptional coactivator with PDZ-binding motif (TAZ) are key downstream components of the Hippo signaling kinase pathway. Isago and colleagues reported that YAP-conditioned knockout in mice caused blockade of branching morphogenesis, whereas a TAZ deficiency gave rise to an emphysema-like phenotype in adult mice (Isago et al. 2020). They also showed that sonic hedgehog, which inhibits the expression of fibroblast growth factor 10 (FGF10) in the mesenchyme, is upregulated by YAP and TAZ in the lung epithelium. FGF10 is considered to be an essential protein in the early stage of lung development (Bellusci et al. 1997). Localized dynamic expression of FGF10 in the mesenchyme adjacent to the distal bud plays an important role in directing outgrowth (Bellusci et al. 1997). Activation of cytoplasmic YAP inhibited FGF10 expression to ensure lung epithelial lineage commitment (Volckaert et al. 2019). Suppression of FGF10 resulted in non-branching trachea (Sekine et al. 1999); in contrast, overexpression of FGF10 brought about aberrant bronchial growth (Isago et al. 2020). Furthermore, the boundary between the airway and distal lung was marked through a nucleocytoplasmic shift of YAP, and it was observed that a YAP deficiency led to SRY-box transcription 9 (SOX9)^pos^ domain expansion and failure to form tube-like airway structures (Mahoney et al. 2014). Those studies suggested that YAP/TAZ play important roles in lung morphogenesis; however, the role of YAP/TAZ in regulating fibroblasts in antenatal inflammation remains unclear. The objective of this study was to investigate regulation of the Hippo signaling pathway in fibroblasts of fetal lungs in a lipopolysaccharide (LPS)-induced inflammation model.

## Materials and methods

### Cell culture and treatment

Human fetal lung IMR-90 fibroblast cells (derived from a 16-week-old female Caucasian fetus) were obtained from the Bioresource Collection and Research Center (Hsinchu, Taiwan). Cells were cultured in 90% minimum essential medium (MEM) with 2 mM l-glutamine and Earle’s balanced salt solution (BSS) adjusted to contain 1.5 g/l sodium bicarbonate, 0.1 mM non-essential amino acids, 1.0 mM sodium pyruvate (Corning, Corning, NY, USA), and 10% fetal bovine serum (FBS) under 5% CO_2_ and 95% relative humidity at 37 °C. Cells were exposed to 10, 30, and 50 μg/ml LPS (*Escherichia coli* O111:B4, Sigma-Aldrich, St. Louis, MO, USA) and control medium (0 μg/ml) for 24 h.

### Fetal lung ex vivo culture and treatment

Pregnant ICR mice were obtained from BioLASCO Taiwan (Taipei, Taiwan) and euthanized at E11.5 (at the pseudoglandular stage). Embryos was collected from the mice followed by lung dissection under a dissecting microscope. All lungs were cultured on Transwell® membranes (Corning) with Biggers, Gwatkin, and Judah (BGJb) medium (Gibco, Grand Island, NY, USA) containing 0.1% FBS, l-ascorbic acid, and primocin (InvivoGen, San Diego, CA, USA). Fetal lungs were exposed to 0, 10, 30, and 50 μg/ml LPS under 5% CO_2_ and 95% relative humidity at 37 °C for 3 days. Medium was collected and replaced everyday. The lung morphology was investigated every 24 h using a Leadview 2000AIO Digital Camera (Taipei, Taiwan). The surface area of the fetal lungs and the number of buds were calculated by ImageJ software (vers. 1.53, National Institutes of Health (NIH), Bethesda, MD, USA) after being normalized to the first day of the experiment.

### Lactate dehydrogenase (LDH)

Supernatants were collected from cells and fetal lung ex vivo culture for an LDH cytotoxicity assay (Donjido Molecular Technology, Rockville, MD, USA). Details of the experimental procedures were in accordance with the manufacturer’s instructions.

### Enzyme-linked immunosorbent assay

IL-6, IL-8 and chemokine (C-X-C motif) ligand 1(CXCL1) levels in supernatants collected from cells and fetal lung culture were examined using enzyme-linked immunosorbent assay (ELISA) kits (ThermoFisher Scientific, Waltham, MA, USA and R&D Systems, Minneapolis, MN, USA). Details of the experimental procedures were in accordance with the manufacturer’s instructions.

### Western blot analysis

Protein from cells and fetal lungs was collected with lysis buffer (Sigma-Aldrich, St. Louis, MO, USA). Samples were electrophoresed in 10% sodium dodecylsulfate polyacrylamide gel electrophoresis (SDS-PAGE) gels and transferred to polyvinylidene difluoride (PVDF) membranes. Membranes were blocked with non-fat dried milk diluted in Tris-buffered saline/Tween-20 (TBST) for 1 h. Samples were incubated with primary antibodies including mouse anti-YAP (1:1000; Proteintech, Rosemont, IL, USA), rabbit anti-p-YAP (1:1000; Abcam, Cambridge, UK), rabbit anti-TAZ (1:1000; Cell Signaling, Danvers, MA, USA), rabbit anti-p-TAZ (1:1000; Cell signaling), rabbit anti-FGF10 (1:1000; Abcam), rabbit anti-FGFR2 (1:1000; Abcam), rabbit anti-SOX2 (1:1000; Cell Signaling), rabbit anti-SOX9 (1:1000; Cell Signaling), rabbit anti-SIRT1 (1:1000; Cell Signaling), rabbit anti p-SIRT1 (1:1000; Signalway Antibody, Greenbelt, MD, USA), and mouse anti-β-actin (1:5000; Proteintech) overnight at 4 °C. After incubation with secondary antibodies for 1 h at room temperature, protein bands were detected with the ChemiDoc™ MP Imaging system (Bio-Rad, Hercules, CA, USA) and quantified by Image-Pro software (vers. 4, Media Cybernetics, Rockville, MD, USA). All data were normalized to the control.

### Immunofluorescence (IF)

Paraffin-embedded fetal lung tissue sections were placed in an oven at 60 °C and rehydrated before staining. Antigen retrieval was done by undergoing heating with citrate buffer (pH 6.0). Bovine serum albumin (BSA, Bionova Scientific, Fremont, CA, USA) with 0.25% Triton X-100 was used for cell permeabilization and 5% BSA was used for blocking at room temperature, followed by incubation with a primary antibody, mouse conjugated fibronectin (1:250; Santa Cruz Biotechnology, Dallas, Tx, USA) for 1.5 h and another primary unconjugated antibody consisting of mouse anti-YAP (1:400; Proteintech), rabbit anti-p-YAP (1:400; Abcam), rabbit anti-TAZ (1:500; Cell Signaling), rabbit anti-p-TAZ (1:500; Cell Signaling), rabbit anti-FGF10 (1:250; BOSTER BIO, Pleasanton, CA, USA), rabbit anti-SOX2 (1:400; Cell Signaling), rabbit anti-SOX9 (1:200; ABGENT, San Diego, CA, USA), rabbit anti-SIRT1 (1:400; Cell Signaling), and rabbit anti p-SIRT1 (1:200; Cell Signaling). A fluorophore-conjugated secondary antibody against the primary antibody was used, and then the sample was covered with mounting medium containing 4′,6-diamidino-2-phenylindole (DAPI, Abcam). Samples were imaged by confocal fluorescence microscopy (TCS SP5, Leica, Wetzlar, Germany) equipped with a camera and imaging software (SPOT Imaging, Sterling-Heights, MI, USA) at 400× magnification. The co-expression mean intensities of YAP, p-YAP, TAZ, p-TAZ, FGF10, SOX2, SOX9, SIRT1, and p-SIRT1 that were fibronectin positive (fibronectin^+^; for identifying fibroblasts) in five different regions were quantified by ImageJ software (NIH) as previously reported (Shihan et al. 2021).

### Immunocytochemistry (ICC)

IMR-90 cells were cultured and then fixed by 2% formaldehyde in PBS for 15 min at room temperature before staining. 0.5% Triton X-100 was used for permeabilization and 5% BSA in PBS was used for blocking at room temperature, followed by incubation with a primary antibody including mouse anti-YAP (1:400; Proteintech), rabbit anti-p-YAP (1:400; Affinity, Melbourne, Victoria, Australia), rabbit anti-TAZ (1:400; Cell Signaling) and rabbit anti-p-TAZ (1:400; Cell Signaling) overnight at 4 °C. A fluorophore-conjugated secondary antibody against the primary antibody was used, and then the sample was covered with mounting medium containing DAPI. Samples were imaged by confocal fluorescence microscopy equipped with a camera and imaging software as the above mentioned at 200× magnification.

### RNA-sequencing

Total RNA was collected from fetal lung tissues in Trizol® reagent (Ambion, Life Technologies, Carlsbad, NY, USA) after exposure to 50 μg/ml LPS and control medium. The purity and quantification were checked using a SimpliNano™-Biochrom Spectrophotometer (Biochrom, Holliston, MA, USA). A Qsep 100 DNA/RNA Analyzer (BiOptic, New Taipei, Taiwan) was used to monitor RNA degradation and integrity. Sequencing libraries were generated using a KAPA messenger (m)RNA HyperPrep Kit (KAPA Biosystems, Roche, Basel, Switzerland) following the manufacturer’s instructions. The original data were obtained through the Illumina NOVAseq 6000 platform. Clean data were obtained by evaluating the parameters including low-quality reads, adaptor contamination, and base qualities. A gene ontology (GO) pathway enrichment analysis of differentially expressed genes (DEGs) was performed using clusterProfiler (vers. 4.4.0). A gene set enrichment analysis (GSEA) was used to identify enriched biological functions and activated pathways from a molecular signature database (MSigDB). A dotplot was created by Rstudio 10.14.

### Statistical analysis

All data are expressed as the mean ± standard deviation (SD). Continuous variables were examined by a one-way analysis of variance (ANOVA) with Tukey’s post-hoc or an unpaired *t*-test. Statistical analyses were performed using GraphPad Prism 7 (San Diego, CA, USA). A *p* value of < 0.05 was considered statistically significant.

## Results

### Cytotoxicity and inflammation

**Fig. 1:**
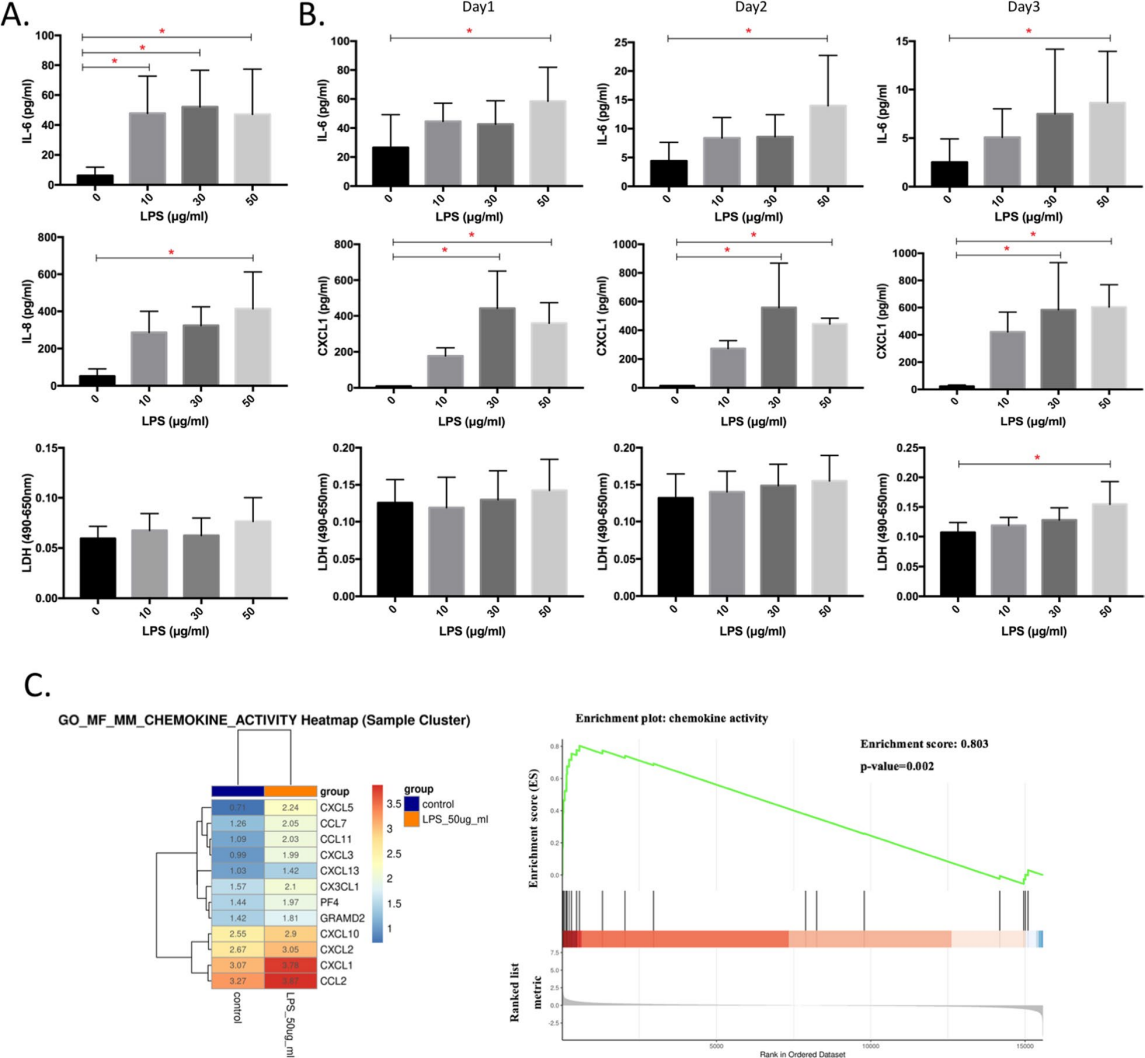
Cytotoxicity and inflammation by lipopolysaccharide (LPS) in IMR-90 cells and ex vivo fetal lungs. **A** Interleukin (IL)-6 (n = 6), IL-8 (n = 3) and lactate dehydrogenase (LDH) (n = 6) in IMR-90 cell supernatants by LPS at 0 (control), 10, 30, and 50 μg/ml. **B** IL-6 (n = 6), CXCL1 (n = 3) and LDH (n = 6) in fetal lung supernatants by LPS at 0, 10, 30, and 50 μg/ml on days 1, 2 and 3. **C** Hierarchical clustering heatmap of significantly expressed gene and gene set enrichment analysis (GSEA) associated with cytokine activity in ex vivo fetal lungs treated by LPS at 0 and 50 μg/ml for 3 days. The activation Z-scores was displayed by the depth of the color (red: upregulation; blue: downregulation). *p < 0.05

Inflammation and cytotoxicity in IMR-90 cells and fetal lung ex vivo culture were shown in Fig. 1. We observed that IL-6 was significantly increased by LPS compared to the control group in IMR-90 cells (*n* = 6, *p* < 0.05; Fig. 1A). IL-8 was significantly increased by 50 μg/ml LPS in IMR-90 (*n* = 3, *p* < 0.05; Fig. 1A). Consistently, IL-6 was increased by 50 μg/ml LPS during the 3 days of exposure in fetal lungs (*p* < 0.05; Fig. 1B). CXCL1 was significantly increased by 30 and 50 μg/ml LPS in fetal lungs (*n* = 3, *p* < 0.05; Fig. 1B). We found that LDH was significantly increased by 50 μg/ml LPS on day 3 after exposure in fetal lungs (*n* = 6, *p* < 0.05; Fig. 1B). We also observed increasing enrichment scores for chemokine activity and genes including CXCL5, CCL7, CCL11, CXCL3, CXCL13, PF4, GRAMD2, CXCL10, CXCL2, CXCL1, CCL2 (*p* < 0.05; Fig. 1C).

### Lung branching morphogenesis

**Fig. 2:**
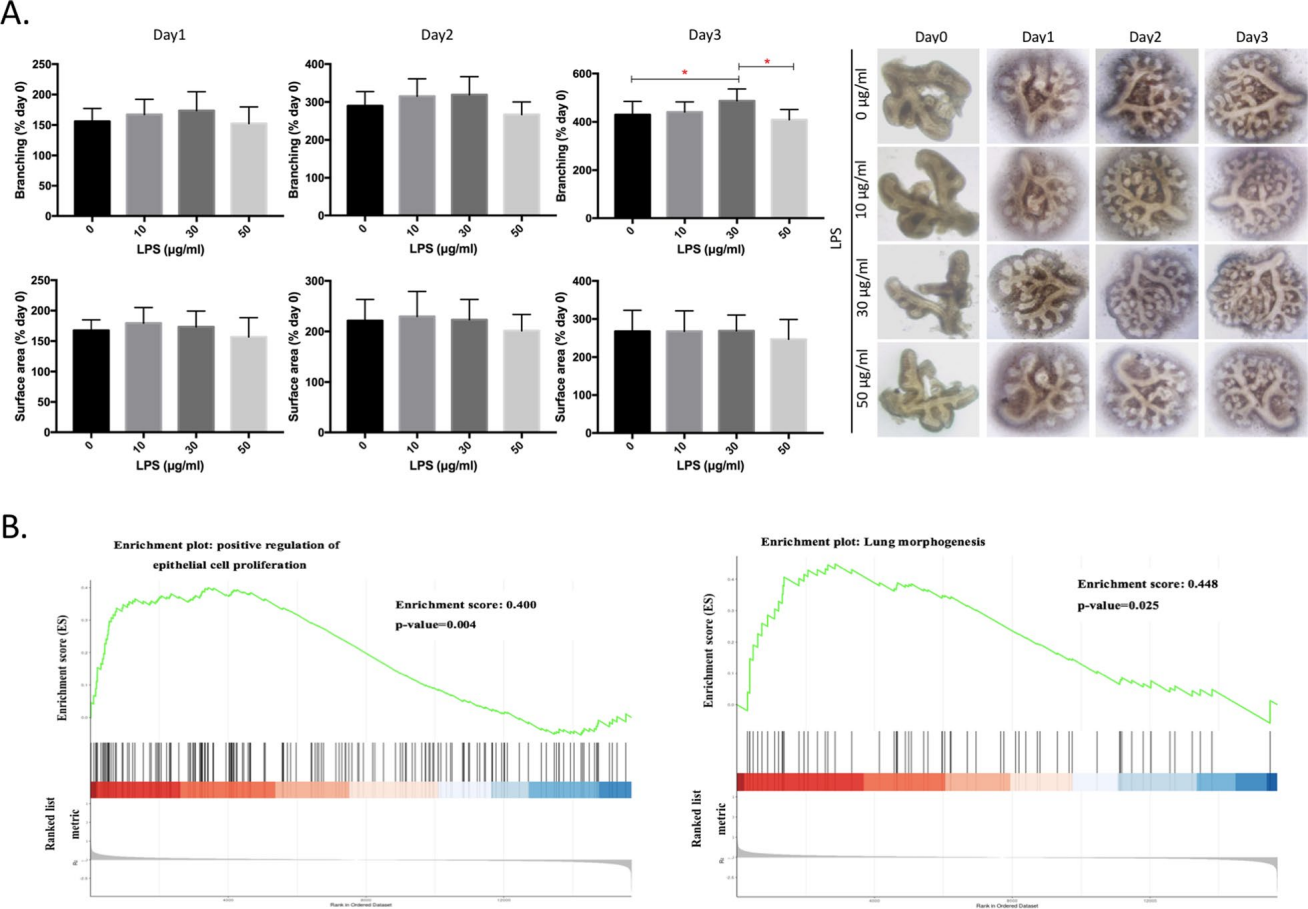
Branching morphogenesis in ex vivo fetal lungs by lipopolysaccharide (LPS). **A** Branching and surface area ratios normalized to day 0 after LPS administration at 0, 10, 30, and 50 μg/ml on days 1, 2, and 3 (*n* = 6). **B** GSEA of enrichment scores by LPS at 0 and 50 μg/ml for 3 days. *p < 0.05

Figure 2 shows the effect of LPS on lung branching morphogenesis in fetal lungs. We observed that lung branching had significantly increased by day 3 after 30 μg/ml LPS exposure (*n* = 6, *p* < 0.05), but the increased level had decreased by day 3 after 50 μg/ml LPS exposure (*n* = 6, *p* < 0.05, Fig. 2A). We also observed increasing enrichment scores for lung epithelial cell proliferation and lung morphogenesis at 50 μg/ml LPS compared to the control (*p* < 0.05, Fig. 2B).

### YAP and TAZ phosphorylation in fibroblasts

**Fig. 3:**
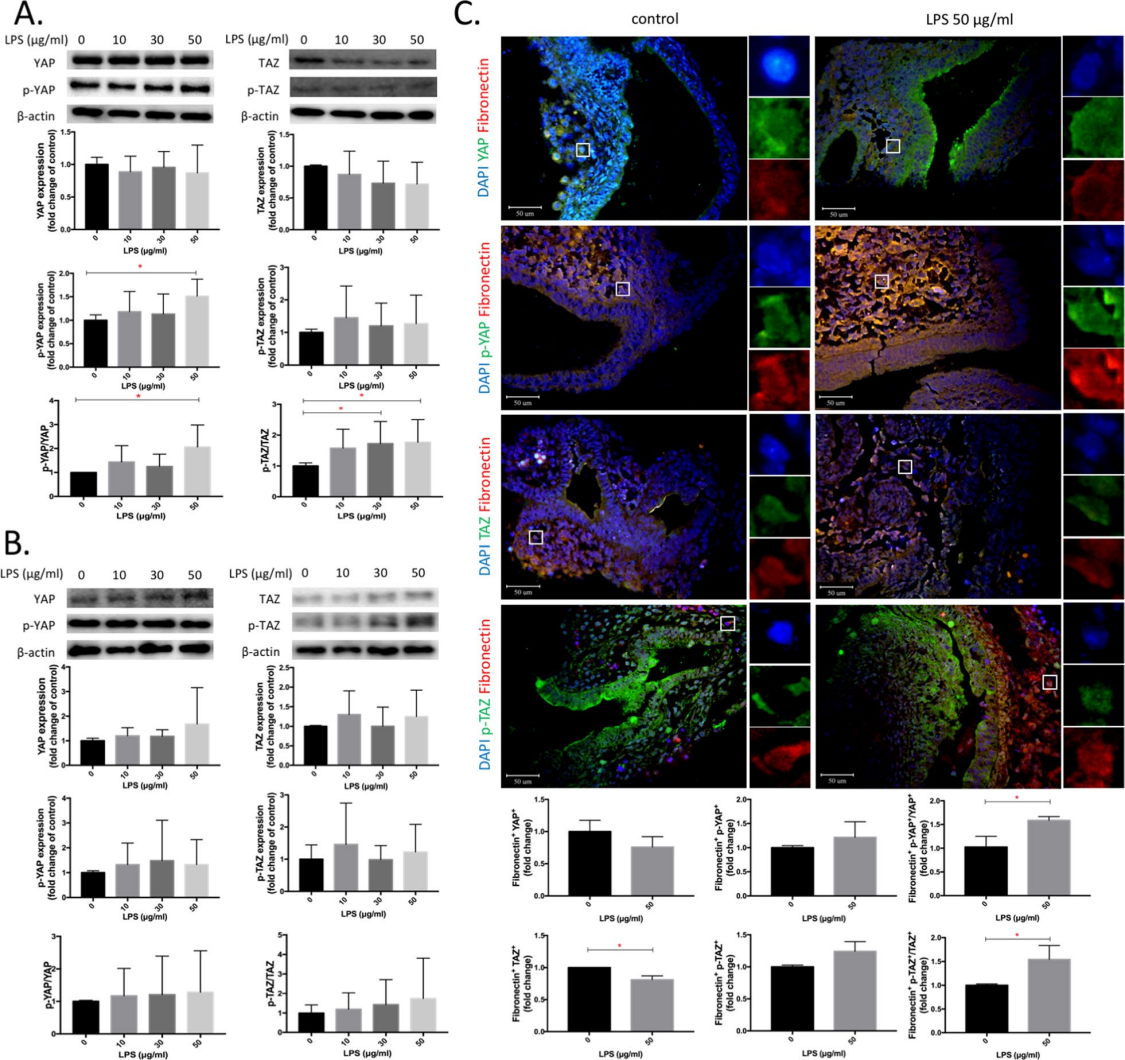
Expression of Yes-associated protein (YAP), phosphorylated (p)-YAP, transcription coactivator with PD2-binding motif (TAZ) and p-TAZ in IMR-90 cells, ex vivo fetal lungs, and fibroblasts of ex vivo fetal lungs by lipopolysaccharide (LPS). **A** Expressions of YAP, p-YAP, p-YAP/YAP, TAZ, p-TAZ, and p-TAZ/TAZ in IMR-90 cells by LPS at 0, 10, 30, and 50 μg/ml for 24 h (*n* = 6). **B** Expressions of YAP, p-YAP, p-YAP/YAP, TAZ, p-TAZ, and p-TAZ/TAZ in whole fetal lungs by LPS at 0, 10, 30, and 50 μg/ml for 3 days (*n* = 6). **C** Expressions of fibronectin^+^ YAP, p-YAP, p-YAP/YAP, TAZ, p-TAZ, and p-TAZ/TAZ of fetal lungs by LPS at 0 and 50 μg/ml for 3 days. DAPI (in blue) marked nuclear staining. Fibronectin (in red). YAP, p-YAP, TAZ, and p-TAZ (in green) (*n* = 3). *p < 0.05

Figure 3 shows YAP and TAZ expressions in fibroblasts in vitro and ex vivo fetal lungs after LPS treatment. We observed that p-YAP and p-YAP/YAP ratios had significantly increased by LPS at 50 μg/ml, and the p-TAZ/TAZ ratios had significantly increased by LPS at 30 and 50 μg/ml compared to the control group in IMR-90 cells (n = 6, *p* < 0.05, Fig. 3A). We further observed decreased YAP and TAZ nuclear expressions with increased p-YAP and p-TAZ cytoplasm expressions after LPS at 30 and 50 μg/ml on IMR-90 cells (Additional file 1: Fig. S1). However, there was no significant difference in YAP or TAZ phosphorylation levels by LPS exposure in ex vivo fetal lungs (Fig. 3B). We next observed increased both fibronectin^+^ p-YAP/YAP and pTAZ/TAZ ratio in fetal lungs at 50 μg/ml LPS, which is consistent with our results in IMR-90 cells (n = 3, *p* < 0.05, Fig. 3C).

### FGF10, SOX2, and SOX9 expressions by fibroblasts

**Fig. 4:**
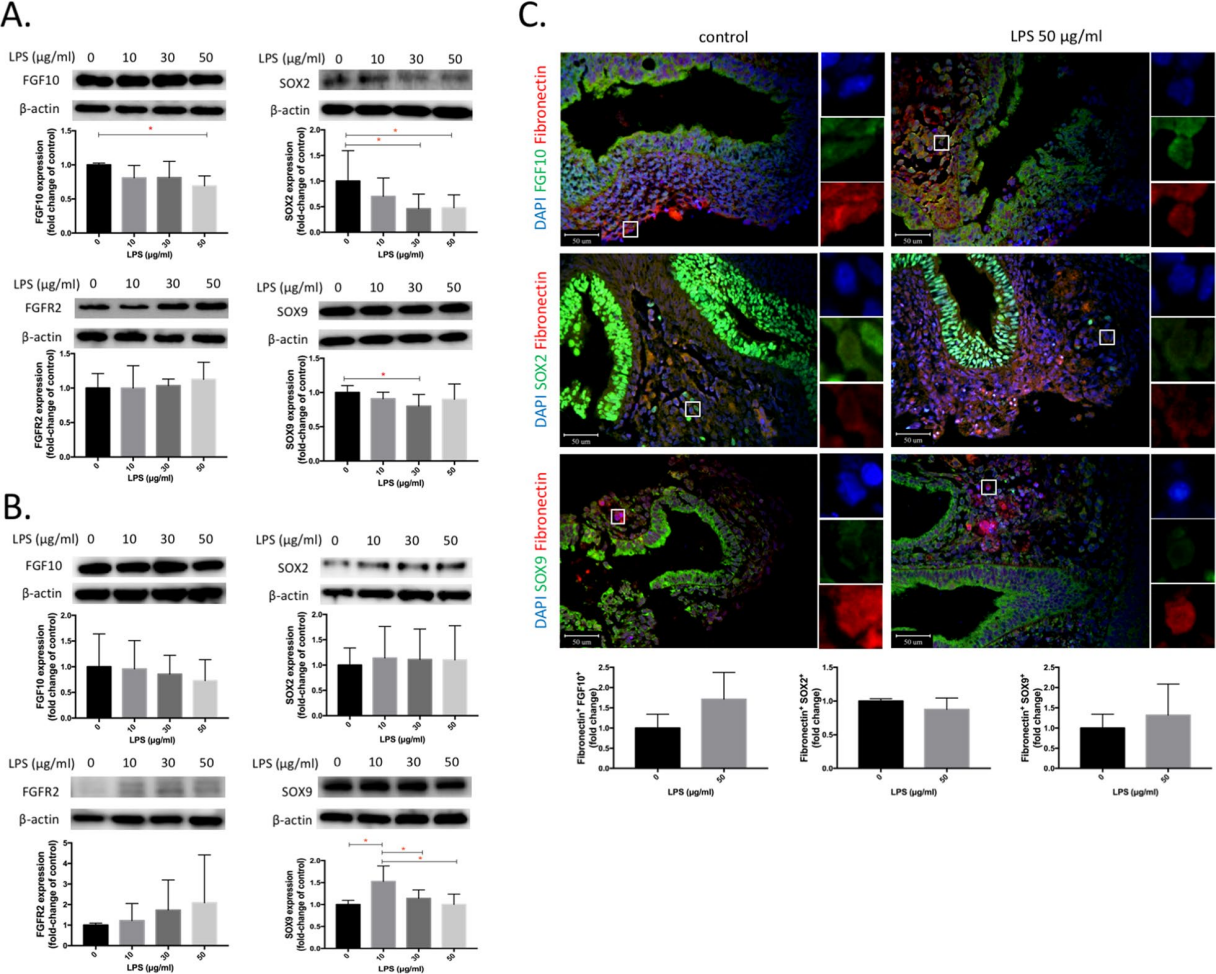
Expressions of fibroblast growth factor 10 (FGF10), FGF receptor 2 (FGFR2), SRY-box transcription factor 2 (SOX2), and SOX9 in IMR-90 cells, ex vivo fetal lungs, and fibroblasts of ex vivo fetal lungs by lipopolysaccharide (LPS). **A** Expressions of FGF10, FGFR2, SOX2, and SOX9 in IMR-90 cells by LPS at 0, 10, 30, and 50 μg/ml for 24 h (*n* = 6). **B** Expressions of FGF10, FGFR2, SOX2, and SOX9 in whole fetal lungs by LPS at 0, 10, 30, and 50 μg/ml for 3 days (*n* = 6). **C** Expressions of fibronectin^+^ FGF10, SOX2, and SOX9 of fetal lungs by LPS at 0 and 50 μg/ml for 3 days. DAPI (in blue). Fibronectin (in red). FGF10, SOX2, and SOX9 (in green) (*n* = 3). *p < 0.05

Figure 4 shows FGF10, FGFR2, SOX2, and SOX9 expressions by fibroblasts in vitro and ex vivo after LPS treatment. We observed that FGF10 significantly decreased by LPS at 50 μg/ml, and SOX9 significantly decreased by LPS at 30 μg/ml compared to the control group. In addition, SOX2 significantly decreased by LPS at 30 and 50 μg/ml in IMR-90 cells (*n* = 6, *p* < 0.05, Fig. 4A). We observed that SOX9 significantly increased by LPS at 10 μg/ml with 3 days of exposure in fetal lungs (*n* = 6, *p* < 0.05, Fig. 4B). We further examined these protein expressions in fibroblasts with fibronectin^+^ in fetal lungs; however, no significant differences were observed (Fig. 4C).

### SIRT1 phosphorylation in fibroblasts

**Fig. 5:**
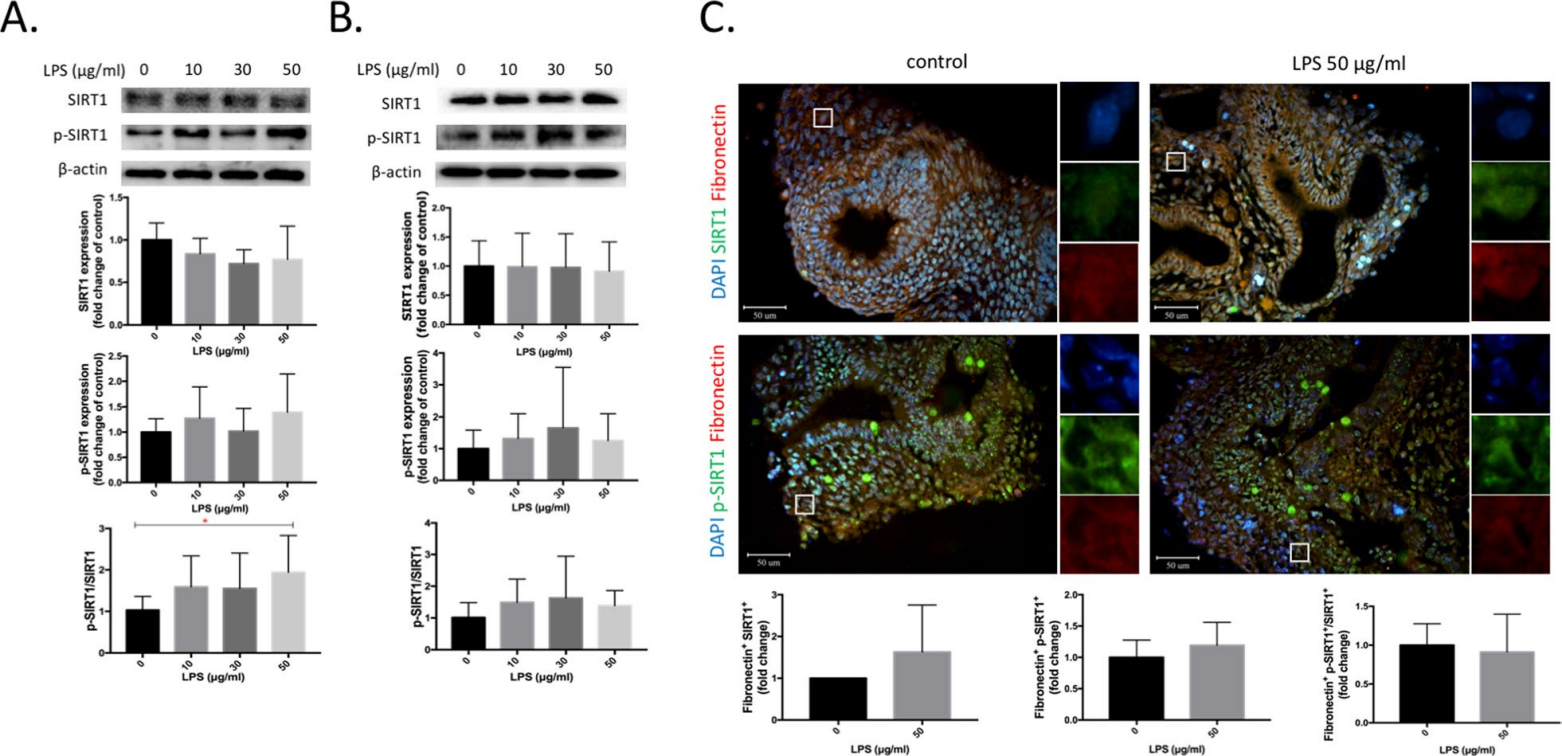
Expressions of sirtuin 1 (SIRT1) and phosphorylated (p)-SIRT1 in IMR-90 cells, ex vivo fetal lungs, and fibroblasts of ex vivo fetal lungs by lipopolysaccharide (LPS). **A** Expressions of SIRT1, p-SIRT1, and p-SIRT1/SIRT1 in IMR-90 cells by LPS at 0, 10, 30, and 50 μg/ml for 24 h (*n* = 6). **B** Expressions of SIRT1, p-SIRT1, and p-SIRT1/SIRT1 in whole fetal lungs by LPS at 0, 10, 30, and 50 μg/ml for 3 days (*n* = 6). **C** Expressions of fibronectin^+^ SIRT1, p-SIRT1, and p-SIRT1/SIRT1 of fetal lungs by LPS at 0 and 50 μg/ml for 3 days. DAPI (in blue). Fibronectin (in red). SIRT1, p-SIRT1 (in green) (*n* = 3). *p < 0.05

Figure 5 shows SIRT1 expression by fibroblasts in vitro and ex vivo after LPS treatment. We observed that the p-SIRT1/SIRT1 ratio significantly increased by LPS at 50 μg/ml compared to the control group in IMR-90 cells (*p* < 0.05, Fig. 5A). However, there was no significant difference in SIRT1 phosphorylation levels by LPS exposure in ex vivo fetal lungs (Fig. 5B). We further examined SIRT1 and p-SIRT1 expressions by fibroblasts with fibronectin^+^ in fetal lungs; however, no significant difference was observed (Fig. 5C).

### Molecular functions and biological pathways of fetal lung

**Fig. 6:**
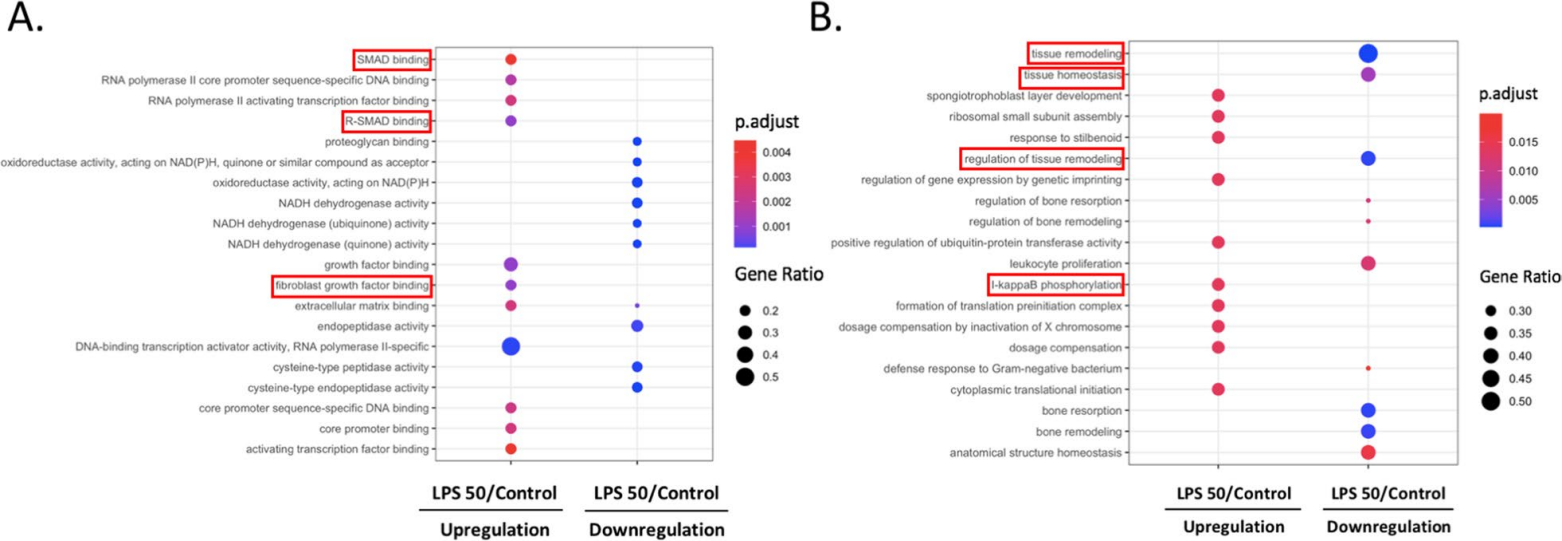
RNA sequencing of ex vivo fetal lungs treated with lipopolysaccharide (LPS). **A** Gene ontology (GO) analysis of molecular functions of genes by LPS at 0 and 50 μg/ml for 3 days. **B** GO analysis of biologic pathways of genes by LPS at 0 and 50 μg/ml for 3 days

Figure 6 shows molecular functions and biological pathways of fetal lungs after 3 days of LPS exposure. As to molecular functions, we observed upregulation of SMAD, (R)-SMAD, core promoter, activating transcription factor, growth factor, and fibroblast growth factor binding, as well as downregulation of proteoglycan binding, oxidoreductase acting on NADPH, NADH dehydrogenase, endopeptidase, and cysteine-type peptidase activity by LPS (*p* < 0.05, Fig. 6A). In addition, we also observed both up- and downregulation of ECM binding by LPS (*p* < 0.05, Fig. 6A). As to biological pathways, upregulation of spongiotrophoblast layer development, ribosomal small subunit assembly, response to stilbenoid, gene expression by genetic printing, IκB phosphorylation, formation of a translation preinitiation complex, dosage compensation, and cytoplasmic translation initiation as well as downregulation of tissue homeostasis and remodeling, bone resorption and remodeling, anatomical structure homeostasis, leukocyte proliferation, and defense of gram-negative bacterium were observed by LPS (*p* < 0.05, Fig. 6B).

## Discussion

The novelty of this study is that we investigated regulation of the Hippo signaling pathway in fibroblasts of fetal lungs in an LPS-induced inflammation model. The significances of our results are as follows: (1) LPS increased inflammation and cytotoxicity, leading to alterations in lung branching morphogenesis, (2) YAP and TAZ phosphorylation in fibroblasts of fetal lungs was activated by LPS, (3) FGF10, SOX2, and SOX9 were downregulated in fibroblasts of fetal lungs by LPS, and (4) SIRT1 phosphorylation in fibroblasts of fetal lungs was upregulated by LPS.

Proinflammatory cytokines of antenatal inflammation are associated with preterm labor (Murthy and Kennea 2007). Increasing evidence indicated that antenatal inflammation causes a systemic inflammatory response, leading to tissue injury in the newborn (Murthy and Kennea 2007). Clinical observations suggested that fetal inflammation increased the risk of BPD (Sarno et al. 2021). In our study, LPS was used to induce antenatal inflammation by increasing IL-6, IL-8 and CXCL1 in vitro and ex vivo. LPS has also been used to induce inflammation in lung epithelial cells (Kim et al. 2012; Hu et al. 2016). A previous study demonstrated that maternal exposure to LPS postponed the alveolarization of the lungs in rats (Cao et al. 2009). Another study reported that chicken embryos in the pseudoglandular stage persistently exposed to LPS exhibited restricted branching morphogenesis of the lungs (Long et al. 2018). Whole mouse fetal lung explants exposed to LPS in the pseudoglandular stage also showed similar results in a dose-dependent manner (Arai et al. 2020). Land and Darakhshan (2004) observed that LPS evoked spontaneous airway branching within a permissive concentration range in the pseudoglandular stage of fetal rat lungs. In our study, we also observed the same phenomenon that the branching ratio increased at low concentrations of LPS, while the increasing level was significantly decreased by a high LPS concentration. Therefore, the results suggested that antenatal inflammation altered lung branching morphogenesis, which could rely on the severity of the inflammatory response.

We observed that LPS increased YAP and TAZ phosphorylation in fibroblasts of fetal lungs. YAP and TAZ are the main downstream mediators of the Hippo pathway, interact with TEA domain (TEAD) family members and activate cell proliferation when translocated into the nucleus, while inducing apoptosis when localized in the cytoplasm (Wang et al. 2017; Lin et al. 2015; Hansen et al. 2015). YAP and TAZ were previously reported to be required during embryonic development to undergo high rates of proliferation (Pocaterra et al. 2020). Previous studies demonstrated that YAP deficiency caused abnormal bronchial morphogenesis, leading to cyst-like structure with thin wall and decreased type 1 alveolar epithelial cells (Mahoney et al. 2014; Nantie et al. 2018; Lin et al. 2017). TAZ deficiency also resulted in emphysema-like changes in lung (Makita et al. 2008; Mitani et al. 2009). Additionally, YAP inhibitor was also reported to delay cell proliferation, epithelial regeneration and recovery of lung injury from LPS (Liu et al. 2020). The impaired regeneration of alveolar epithelial due to lack of YAP/TAZ was paralleled with NF-κB proinflammatory signal pathway, which inhibited lung branching and epithelial growth (LaCanna et al. 2019; Muraoka et al. 2000). Lung branching morphogenesis requires epithelial-mesenchymal interactions (Hogan and Yingling 1998). It was proven that two primary lung buds without mesenchyme stops processing branching (Wessells 1970). Our findings suggest that regulation of the Hippo pathway in the surrounding mesenchyme, such as fibroblasts, occurred by LPS in fetal lungs. Together, this suggested that the phosphorylation of YAP and TAZ in fibroblasts by LPS could be relevant to abnormal branching under inflammation.

Next, we observed decreased expressions of FGF10, SOX2, and SOX9 in fibroblasts after LPS treatment. FGF10 is secreted by fibroblasts of the mesenchyme and guides lung epithelial branching (Yin and Ornitz 2020). A previous study showed that lung hypoplasia with esophageal atresia was caused by downregulating FGF10 signaling in vivo (Wang et al. 2018a). Another study reported that LPS affected branching morphogenesis via decreasing *FGF10* gene expression (Muratore et al. 2009). Consistent with our results, high dose LPS significant reduced FGF10 expression and might be negatively associated with lung branching morphogenesis (Benjamin et al. 2010). SOX2 and SOX9 play essential roles in the proliferation and differentiation of the proximal and distal lung epithelium, respectively (Danopoulos et al. 2018). SOX2 is restricted to the proximal lung epithelium during lung development (Gontan et al. 2008). A previous study demonstrated that the loss of SOX2 led to an immature secretory and ciliated system in conducting airways (Tompkins et al. 2011). SOX9 is expressed by the distal lung epithelium as well as in the mesenchyme surrounding the proximal lung (Fernandes-Silva et al. 2017). It was found to promote branching morphogenesis by regulating not merely the balance between distal epithelium differentiation and proliferation but the ECM as well (Rockich et al. 2013). As a consequence, decreasing expressions of FGF10, SOX2 and SOX9 by fibroblasts with LPS treatment could be associated with branching defects under inflammation (Mia and Singh 2022).

SIRT1, a NAD^+^-dependent deacetylase, is an anti-apoptotic factor and increases resistance to oxidative damage in mammalian cells (Alcendor et al. 2007). In our study, we found increased SIRT1 phosphorylation in fibroblasts of fetal lungs after LPS treatment. Previous study showed that SIRT1 phosphorylation by administration of dexmedetomidine significantly reduce sepsis-induced lung injury in rat model (Wang et al. 2020). Another study showed that SIRT1 phosphorylation was associated with anti-oxidative and anti-inflammation on endothelial cells (Kitada et al. 2016). SIRT1 phosphorylation can attenuate drug induced apoptosis and coactivated heat shock factor 1, which was responsible for activate the protective factors in response to stress (Wang et al. 2018b; Monteiro and Cano 2011). Consistently, SIRT1 overexpression protected normal human fibroblast IMR-90 cells from H_2_O_2_ injury, with loss of SIRT1 phosphorylation resulted in decreased activity and loss of survivability (Luo et al. 2001; Sasaki et al. 2008). SIRT1 was also found to alleviate LPS-induced lung injury in animal models by decreasing the endothelial permeability (Fu et al. 2019). Taken together, SIRT1 phosphorylation in fibroblasts under LPS-induced inflammation might be associated with a protective mechanism to mitigate injury from inflammation.

Based on RNA-sequencing results, we observed that LPS exposure upregulated the binding ability including SMAD and FGF. The *SMAD* gene is involved in the TGF-β and BMP signaling pathway (Zhao et al. 1998). SMAD-2, -3, and -7 are responsible for *TFG-β* gene expression, and several studies demonstrated that the addition of exogenous TGF-β inhibited lung branching (Warburton et al. 2003). A BMP-specific receptor regulates Smads (R-Smads) including SMAD-1, -5, and -8 and transduces the BMP4 ligand into nuclei (Massagué and Chen 2000). Overexpression of BMP4 promoted by the SP-C enhancer caused abnormalities in the lungs with cystic terminal sacs and inhibition of epithelial cell proliferation (Bellusci et al. 1996). Similar results were also observed in localized FGF10 overexpression in fetal rat lungs (Gonzaga et al. 2008). In addition, we also observed that LPS upregulated the phosphorylation of IκB. Phosphorylation of IκB activates nuclear factor (NF)-κB, which is regarded as a proinflammatory signal pathway (Karin and Ben-Neriah 2000; Lawrence 2009). A previous study found that activation of NF-κB in the mesenchyme inhibited lung branching and epithelial growth (Muraoka et al. 2000). Consistently, the downregulation of tissue homeostasis and remodelling by LPS were observed. Hippo signaling pathway was previously reported to be involved in tissue homeostasis and remodeling, with delayed cell proliferation, epithelial regeneration and lung injury recovery from LPS by YAP inhibitor (Liu et al. 2020; Mia and Singh 2022). The impaired regeneration of alveolar epithelial due to lack of YAP/TAZ was accompanied with failure in terminating NF-κB proinflammatory signal pathway (LaCanna et al. 2019). Taken together, abnormal branching was confirmed by the results from RNA-sequencing under LPS-induced inflammation in fetal lungs.

**Fig. 7:**
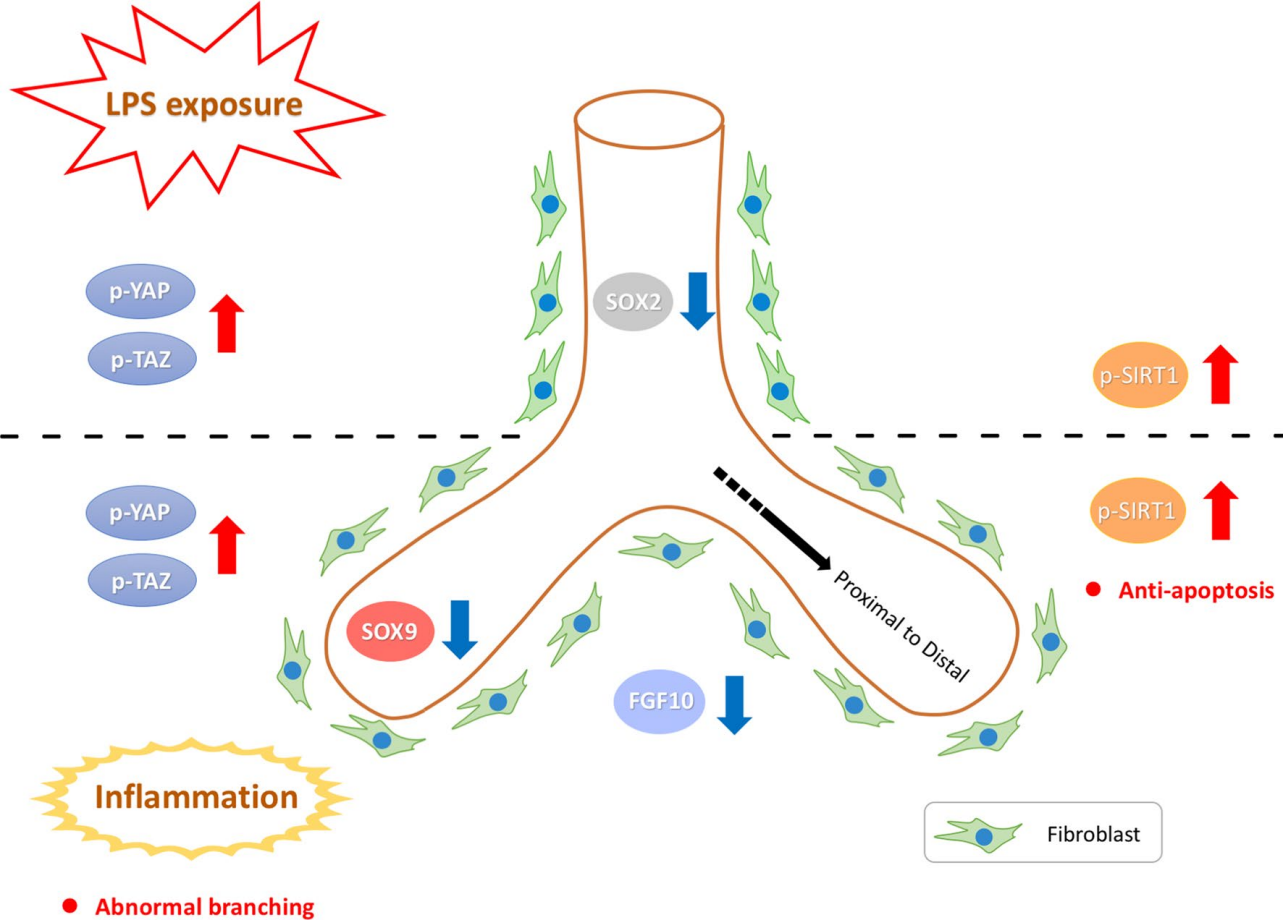
Summary. High dose LPS exposure induced the inflammatory response and led to YAP/TAZ phosphorylation in fibroblast of fetal lungs in pseudoglandular stage. FGF10 secreted by mesenchyme, proximal and distal airway markers SOX2 and SOX9 expression decreased branching and caused abnormal branching. Phosphorylation of anti-apoptotic factor SIRT1 after exposure to LPS might be associated with mitigating the injury from inflammation

These are the first data to our knowledge showing dysregulation of the Hippo signaling pathway in fibroblasts of fetal lungs in an LPS-induced inflammation model, as depicted in a summary schematic (Fig. 7). There are some limitations to our study. Future studies should be conducted to clarify the biphasic model of branching morphogenesis in different developing stages of the lungs, confirm the senescence and the relationship between phosphorylation of SIRT1 and its activity in our study. In addition, future study by performing block and rescue method will be performed to confirm YAP/TAZ regulated lung branching.

## Conclusions

In conclusion, our results suggested that abnormal branching occurred under LPS-induced inflammation, which involved regulation of the Hippo pathway via YAP/TAZ phosphorylation in fibroblasts of fetal lungs. This study showed the importance of understanding the role of the Hippo pathway in the surrounding mesenchyme of fetal lungs under inflammation. Clinically, it could help us understand the relationship between antenatal inflammation and lung branching.

## Supplementary Information

**Additional file 1: Figure S1.** Representative immunocytochemistry staining of YAP, phosphorylated (p)-YAP, TAZ, and p-TAZ expressions in IMR-90 cells by lipopolysaccharide (LPS) at 0, 10, 30 and 50 μg/mL for 24 h. YAP and TAZ were stained in red, p-YAP and p-TAZ were stained in green, and nuclear staining were marked by DAPI in blue.

## Data Availability

The datasets used and/or analyzed during the current study are available from the corresponding author on reasonable request.
