# Supplementary material for: Alteration in branching morphogenesis via YAP/TAZ in fibroblasts of fetal lungs in an LPS-induced inflammation model
Source: Mol Med. 2023 Jan 30;29:16. doi: 10.1186/s10020-023-00613-w (PMC9887856; doi:10.1186/s10020-023-00613-w)
Supplement: Supplementary file 1 — Additional file 1: Figure S1. Representative immunocytochemistry staining of YAP, phosphorylated (p)-YAP, TAZ, and p-TAZ expressions in IMR-90 cells by lipopolysaccharide (LPS) at 0, 10, 30 and 50 μg/mL for 24 h. YAP and TAZ were stained in red, p-YAP and p-TAZ were stained in green, and nuclear staining were marked by DAPI in blue. [file 10020_2023_613_MOESM1_ESM.docx]

**Additional file 1**

**Alteration in branching morphogenesis via YAP/TAZ in fibroblasts of fetal lungs in an LPS-induced inflammation model**

Hung-Shuo Ko^1^, Vincent Laiman^2,3^, Po-Nien Tsao^4^, Chung-Ming Chen^5,6^, Hsiao-Chi Chuang^7,8,9,10*^

^1^School of Medicine, College of Medicine, Taipei Medical University, Taipei, Taiwan

^2^International Ph.D. Program in Medicine, College of Medicine, Taipei Medical University, Taipei, Taiwan

^3^Department of Anatomical Pathology, Faculty of Medicine, Public Health, and Nursing, Universitas Gadjah Mada – Dr. Sardjito Hospital, Yogyakarta, Indonesia

^4^Department of Pediatrics, National Taiwan University Hospital, Taipei, Taiwan

^5^Department of Pediatrics, Taipei Medical University Hospital, Taipei, Taiwan

^6^Department of Pediatrics, School of Medicine, College of Medicine, Taipei Medical University, Taipei, Taiwan

^7^School of Respiratory Therapy, College of Medicine, Taipei Medical University, Taipei, Taiwan

^8^Division of Pulmonary Medicine, Department of Internal Medicine, Shuang Ho Hospital, Taipei Medical University, New Taipei City, Taiwan

^9^Cell Physiology and Molecular Image Research Center, Wan Fang Hospital, Taipei Medical University, Taipei, Taiwan

^10^Graduate Institute of Medical Sciences, College of Medicine, Taipei Medical University, Taipei, Taiwan

**Running Head:** YAP/TAZ regulates branching morphogenesis of fibroblasts

**Word count:** 3355 [excluding abstract (265 words) and references]; [6] figures.

***Corresponding Author**

*Hsiao-Chi Chuang, PhD*

Inhalation Toxicology Research Lab (ITRL), School of Respiratory Therapy, College of Medicine, Taipei Medical University, 250 Wuxing Street, Taipei 11031, Taiwan.

Telephone: +886-2-27361661 ext. 3512. Fax: +886-2-27391143. E-mail: [chuanghc@tmu.edu.tw](mailto:chuanghc@tmu.edu.tw)

**Figure and legend**

**
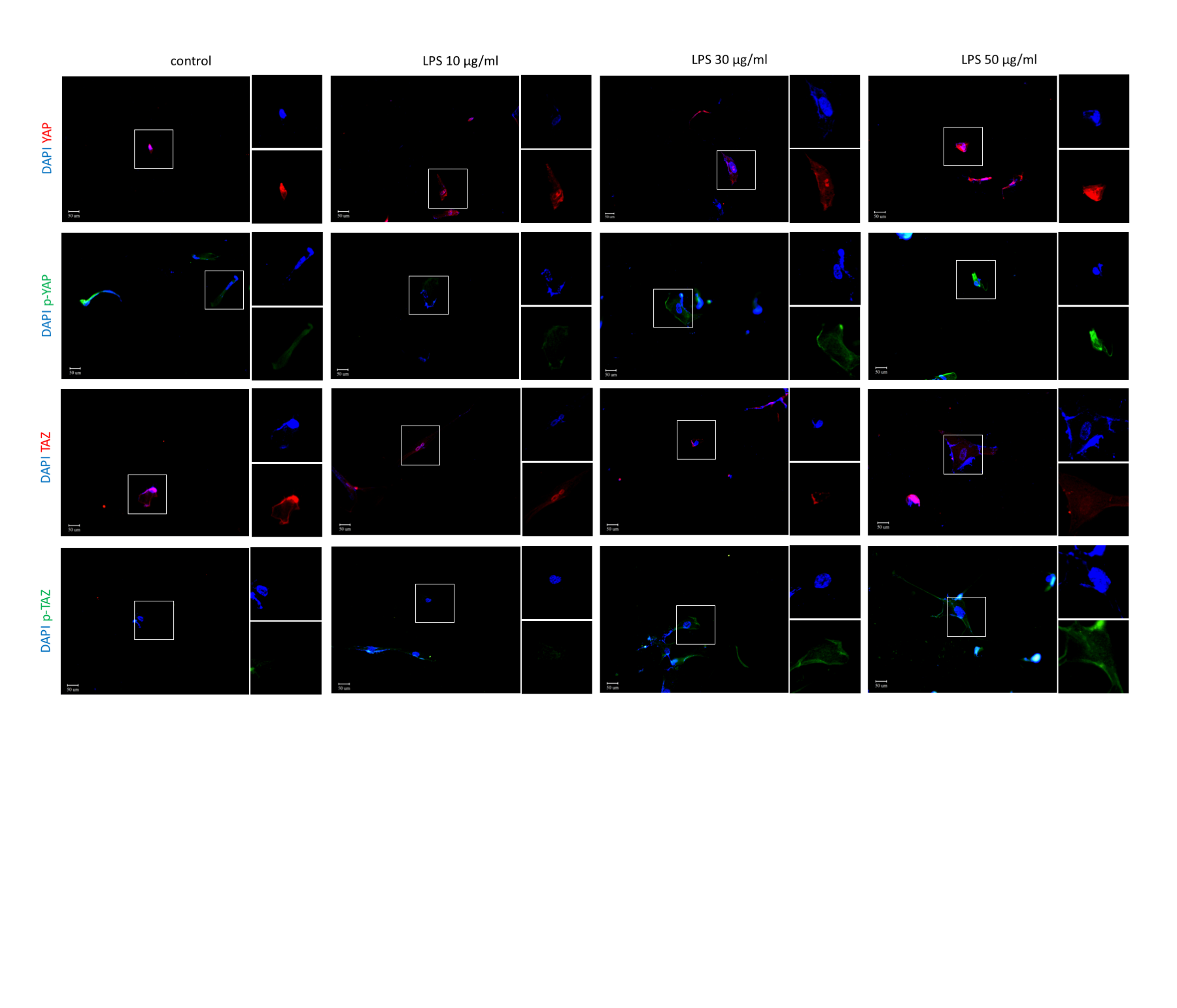
**

**Figure S1.** Representative immunocytochemistry staining of YAP, phosphorylated (p)-YAP, TAZ, and p-TAZ expressions in IMR-90 cells by lipopolysaccharide (LPS) at 0, 10, 30 and 50 μg/mL for 24 hours. YAP and TAZ were stained in red, p-YAP and p-TAZ were stained in green, and nuclear staining were marked by DAPI in blue.
